# Nanodots of Transition Metal Sulfides, Carbonates, and Oxides Obtained Through Spontaneous Co-Precipitation with Silica

**DOI:** 10.3390/nano14242054

**Published:** 2024-12-23

**Authors:** Bastian Rödig, Diana Funkner, Thomas Frank, Ulrich Schürmann, Julian Rieder, Lorenz Kienle, Werner Kunz, Matthias Kellermeier

**Affiliations:** 1 Institute of Physical and Theoretical Chemistry, University of Regensburg, D-93040 Regensburg, Germany; diana.funkner@ur.de (D.F.); thomas.frank82@web.de (T.F.);; 2 Faculty of Engineering, Kiel University, Kaiserstr. 2, D-24143 Kiel, Germany; usc@tf.uni-kiel.de (U.S.); lk@tf.uni-kiel.de (L.K.); 3 Material Science, BASF SE, RGA/BM–B007, Carl-Bosch-Str. 38, D-67056 Ludwigshafen, Germany

**Keywords:** nanodots, transition metal oxides, transition metal sulfides, silica, co-precipitation, core–shell particles, self-assembly

## Abstract

The controlled formation and stabilization of nanoparticles is of fundamental relevance for materials science and key to many modern technologies. Common synthetic strategies to arrest growth at small sizes and prevent undesired particle agglomeration often rely on the use of organic additives and require non-aqueous media and/or high temperatures, all of which appear critical with respect to production costs, safety, and sustainability. In the present work, we demonstrate a simple one-pot process in water under ambient conditions that can produce particles of various transition metal carbonates and sulfides with sizes of only a few nanometers embedded in a silica shell, similar to particles derived from more elaborate synthesis routes, like the sol–gel process. To this end, solutions of soluble salts of metal cations (e.g., chlorides) and the respective anions (e.g., sodium carbonate or sulfide) are mixed in the presence of different amounts of sodium silicate at elevated pH levels. Upon mixing, metal carbonate/sulfide particles nucleate, and their subsequent growth causes a sensible decrease of pH in the vicinity. Dissolved silicate species respond to this local acidification by condensation reactions, which eventually lead to the formation of amorphous silica layers that encapsulate the metal carbonate/sulfide cores and, thus, effectively inhibit any further growth. The as-obtained carbonate nanodots can readily be converted into the corresponding metal oxides by secondary thermal treatment, during which their nanometric size is maintained. Although the described method clearly requires optimization towards actual applications, the results of this study highlight the potential of bottom-up self-assembly for the synthesis of functional nanoparticles at mild conditions.

## 1. Introduction

Nanoparticles continue to be an important type of material for a broad range of applications, including catalysis [1], electronics [2], energy storage [3], (bio-)sensors [4], functional coatings [5,6], and many more [7]. While a variety of different approaches to the synthesis of defined nanoparticles have been developed (such as sol–gel chemistry [8], hydrothermal [9] and solvothermal [10] processes, or mechanochemical concepts [11]), it is still a challenge to control their size, composition, structure (e.g., crystalline vs. amorphous) and surface functionalities (which are key to interactions and, thus, final properties). In addition, nanoparticle systems inherently suffer from their tendency to agglomerate, and hence, means to achieve sufficient colloidal stabilization need to be found [12]. In many cases, organic additives like surfactants, polymers, or small molecules are used to influence the size and shape of the forming nanoparticles and improve their colloidal stability in the targeted formulation [13,14,15,16]. However, in view of actual applications, these additives often have the disadvantage of high costs related to their synthesis or purification, possible contamination of the environment (since the additive can be released from the nanoparticle at some point), and/or the fact that controlled nanoparticle formation only occurs under particular conditions [17]. Given these limitations, robust methods based on self-organizing simple and harmless components at mild conditions appear highly desirable.

In this context, one particularly interesting but rather rarely considered case is the inorganic–inorganic route to self-assembled nanostructures, most notably the spontaneous stabilization of nanoparticles using silica. Indeed, there is a vast body of literature on coating various types of nanomaterials with silica to improve stability and achieve other beneficial effects [18,19,20,21,22]. However, instead of depositing a silica shell around preformed nanoparticles in a secondary step, silicate species dissolved in aqueous solutions can also be used as primary “capping agents” to preserve growing particles at nanometric dimensions by chemically coupled co-precipitation [23,24]. For this spontaneous process to occur, the material in question needs to show inverse trends in solubility as a function of pH compared to silica, and its constituent ions (or molecules) should exist in a partially protonated state under the chosen synthesis conditions [25]. One prominent example meeting these criteria is the precipitation of alkaline-earth metal cations (M^2+^ = Ca^2+^ or Ba^2+^) with carbonate anions from water at moderately alkaline pH levels in a typical range of 9–11 [26,27], which can be written as follows:

(1) $${\mathrm{HCO}}_{3}^{-}\;⇆{\;\mathrm{CO}}_{3}^{2-}{+H}^{+}\;\overset{M^{2+}}{\to }{\;\mathrm{MCO}}_{3}↓$$

The release of protons caused by the re-establishment of the carbonate/bicarbonate equilibrium results in a decrease of the local pH near growing metal carbonate surfaces, which in turn triggers polycondensation of dissolved silicate species according to:

(2) $${\text{-}\mathrm{Si}\text{-}O}^{-}{+H}^{+}\;⇄\;\text{-}\mathrm{Si}\text{-}\mathrm{OH}+\mathrm{HO}\text{-}\mathrm{Si}\text{-}\;\overset{-H_{2}O}{\to }\;\text{-}\mathrm{Si}\text{-}O\text{-}\mathrm{Si}\text{-}\;\to \to \;{“\mathrm{SiO}}_{2}”↓$$

**Scheme 1:**
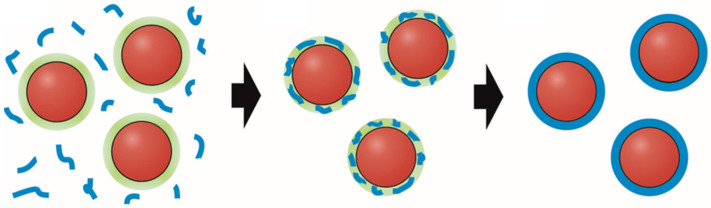
Spontaneous coating of metal carbonate particles (red circles) with shells of amorphous silica (blue rims) as a consequence of local pH gradients (green halos) in alkaline solutions, caused by bicarbonate dissociation and triggering the polycondensation of dissolved silicate species (blue shreds) in the immediate vicinity of growing particles. Redrawn according to the concept introduced in ref. [26].

This pH-based coupling of the chemistries of carbonate and silicate eventually leads to the spontaneous coating of MCO_3_ particles with SiO_2_ shells, as illustrated by Scheme 1. The response of silicate species to changes in the local microenvironment around forming carbonate particles has three important consequences: first, growth of the cores is arrested soon after nucleation, such that nanoparticles of tunable sizes are obtained; second, local polycondensation yields continuous layers of amorphous silica around the carbonate cores in the same step, with thicknesses that can likewise be readily tuned by proper choice of the bulk silicate concentration and/or the pH of the solution; third, the surface energy of the formed nanodots is minimized stabilizing them at very small sizes [26,27,28].

In the case of alkaline-earth metal carbonates, this concept of the spontaneous stabilization of nanodots by a coprecipitation of both the core and the stabilizing silica shell could readily be realized by simple mixing of calcium or barium chloride solutions (at typical concentrations of 10 mM) with alkaline sols containing sodium silicate. At silicate concentrations of up to 2000 ppm (33.3 mM) in the final mixture, nanoparticles of amorphous CaCO_3_ cores with typical sizes of 30–150 nm and silica shells with thicknesses in the range of 2–10 nm were obtained [26], whereas SiO_2_-BaCO_3_-SiO_2_ core–shell-shell structures formed when barium cations were used under similar conditions [27]. Subsequent studies performed at higher silicate concentrations (≥3000 ppm as SiO_2_) have further shown that CaCO_3_ clusters of only a few nanometers in diameter could be stabilized by the same approach [28], confirming the notion that silica can effectively capture the earliest precursors of inorganic crystallization processes.

Regarding the model of chemically coupled spontaneous co-precipitation described above, there is no apparent reason why the underlying interactions should be limited to alkaline-earth metal cations. Likewise, anions other than carbonate may engage in chemical coupling with silicate, provided that they exist at least partially in a protonated state under the chosen conditions. Indeed, both of these hypotheses have been sustained by the results of a recent study, in which silica-coated cadmium sulfide (as well as zinc sulfide and cadmium selenide) quantum dots were successfully synthesized by co-precipitation from aqueous solutions at ambient temperature [29]. In this case, the reaction leading to nucleation and growth of the metal sulfide cores can be written as:

(3) $${\mathrm{HS}}^{-}\;⇆{\;S}^{2-}{+H}^{+}\;\overset{M^{2+}}{\to }\mathrm{MS}\;↓$$

Building upon these findings, the goal of the present work was to test the generality of the concept and extend the range of nanoparticles accessible via spontaneous stabilization with silica by self-assembly. To this end, various transition metal salts were precipitated with carbonate or sulfide in the presence of silicate, and the outcome of the reaction was characterized using transmission electron microscopy (TEM). It will be shown that despite the simplicity of the approach, well-defined metal carbonate and sulfide particles with diameters of less than 5 nm (hereinafter referred to as “nanodots”) can be obtained and, in the case of carbonates, converted into corresponding oxides by secondary thermal treatment. Overall, the described procedures devise a straightforward and sustainable strategy towards the synthesis of functional nanomaterials with potential for applications in the areas of catalysis, biomedicine, and beyond.

## 2. Materials and Methods

*Chemicals*: The following substances were used as received without further purification: cadmium chloride (CdCl_2_; Sigma-Aldrich, St. Louis, MO, USA, 99.99%), cobalt chloride hexahydrate (CoCl_2_·6H_2_O; Sigma-Aldrich, St. Louis, MO, USA,, 98%), copper chloride dihydrate (CuCl_2_·2H_2_O; Riedel-de Ha$\overset{..}{e}$n, Seelze, Germany, ≥99%), manganese chloride tetrahydrate (MnCl_2_·4H_2_O; Sigma-Aldrich, St. Louis, MO, USA, ≥99%), nickel chloride hexahydrate (NiCl_2_·6H_2_O; Merck, Darmstadt, Germnnay, ≥98%), zinc chloride (ZnCl_2_; Merck, Darmstadt, Germany, ≥98%), ammonium carbonate ((NH_4_)_2_CO_3_; AppliChem, Darmstadt, Germany, analytical grade), sodium carbonate (Na_2_CO_3_; Riedel-de Ha$\overset{..}{e}$n, Seelze, Germany, ≥99.8%), sodium sulfide trihydrate (Na_2_S·3H_2_O; VWR International, Radnor, PA, USA, analytical grade), sodium silicate solution (“water glass”, containing 27% SiO_2_ and 14% NaOH; Sigma-Aldrich, St. Louis, MO, USA, reagent grade), tetraethylorthosilicate (TEOS; Sigma-Aldrich, St. Louis, MO, USA, 98%), sodium chloride (NaCl; Carl Roth, Karlsruhe, Germany, ≥99.8%), sodium hydroxide (NaOH; Sigma-Aldrich, St. Louis, MO, USA, ≥98%), and hydrochloric acid (32% HCl in water; Sigma-Aldrich, St. Louis, MO, USA, analytical grade). All solutions and dilutions were prepared using water taken from a Millipore system.

*Synthesis of Nanoparticles*: First, aqueous solutions containing 0–33.3 mM (equal to 0–2000 ppm) sodium silicate were prepared by diluting commercial sodium silicate solution (“water glass”) or hydrolyzing tetraethylorthosilicate with sodium hydroxide at pH 11, using plastic beakers to avoid potential covalent attachment to the surface of glass vessels by silica condensation reactions. Then, 10–20 mM of sodium sulfide, sodium carbonate, or ammonium carbonate were dissolved in the SIlicate solution. To 50 mL of the resulting mixtures, the same volume of a solution of divalent transition metal chloride was quickly added under vigorous stirring (600 rpm) at room temperature. Usually, the amounts of transition metal (M) cation and sulfide or carbonate were chosen to be equimolar, while the silicate content was varied. Typical final concentrations, thus, were 5–10 mM MS/MCO_3_ and 0–16.7 mM SiO_2_. Immediately after mixing, colored suspensions of more or less finely dispersed precipitates were obtained. After stirring for 5 min, the formed particles were separated from dissolved salt (mainly NaCl or NH_4_Cl) by centrifugation in a Sigma 3-18KS centrifuge at 4700 rpm (4248 g) for 5 min at room temperature. If separation was not possible under these conditions, a few drops of 5 M sodium chloride solution were added before centrifugation. The sedimented material was washed three times with water to further remove any residues of sodium/ammonium chloride unreacted residues. Finally, the purified particles were re-dispersed in water using an ultrasonic processor (Hielscher UP200Ht). To convert the as-formed silica-coated transition metal carbonates into oxides, corresponding dispersions were lyophilized to obtain a dry powder, which was calcined in a Thermicon T oven from Heraeus under atmospheric pressure. The following calcination temperatures and times were applied, following the procedures described in the literature [30,31,32,33,34]: CdCO_3_—500 °C for 2 h, CoCO_3_—500 °C for 3 h, CuCO_3_—400 °C for 2 h, MnCO_3_—400 °C for 4 h, NiCO_3_—500 °C for 2 h, ZnCO_3_—600 °C for 2 h.

*Dynamic Light Scattering (DLS)*: Information about the size of the as-formed nanoparticles and their aggregates was studied using dynamic light scattering measurements. To this end, 0.5 mL of the re-dispersed samples were diluted with 2.0 mL water, filtered through 0.45 μm cellulose acetate membranes, and transferred into dust-free cylindrical borosilicate glass cells of 10 mm outer diameter. The sealed DLS tubes were placed in the temperature-controlled toluene bath of a CGS-3 Compact Goniometer System from ALV, which was equipped with an ALV/LSE-5004 Multiple Tau Digital Correlator and a vertically polarized 22 mW He-Ne laser (λ = 632.8 nm). Measurements were performed at 25 °C and a scattering angle of 90°. The sampling time for sulfide and carbonate nanoparticles was 300 s. As suspensions of oxide nanoparticles were less stable, the sampling time for these systems was reduced to 60 s. From the collected data, the mean intensities of the scattered light and the time-dependent self-correlation functions were determined. The average hydrodynamic diameters of the scattering species were extracted via a second-order cumulant analysis [35] and the CONTIN algorithm [36]. As many of the studied systems showed rather high polydispersity, the derived apparent sizes should be considered as rough estimates of the dimensions of nanostructures occurring in solution.

*Transmission Electron Microscopy (TEM)*: To elucidate the nanostructure of the formed precipitates, the obtained dispersions were diluted with water to particle volume densities low enough to enable meaningful TEM analyses. For this purpose, 20 µL dilute samples were placed on copper grids (400 mesh × 62 μm pitch) that were coated with a thin layer of graphite (using a Carbon Coater 208carbon from Cressington) and hydrophilized via treatment in a PDC-3XG plasma cleaner from Harrick Plasma. After soaking for 20 s, excess liquid was removed by blotting with filter paper. The fixed specimen on the grid was washed with 20 μL water and then dried again by blotting. Finally, the grid was left to dry in a closed Petri dish on filter paper for at least 24 h before it was transferred into the electron microscope using a Gatan cryo-holder. TEM studies were performed on a CM12 microscope from FEI/Philips, equipped with a tungsten cathode operating at an accelerating voltage of 120 kV. Images were taken with a TVIPS digital camera and processed using the software EMMenu4 4. Electron diffraction patterns were measured on a Tecnai F30 G^2^ STwin from FEI (300 kV) equipped with an EDX detector (Si/Li, EDAX). ADF-STEM micrographs and EDX elemental mapping were conducted on a probe Cs-corrected JEM-ARM200F NEOARM from JEOL (200 kV) equipped with two front and entry side positioned silicon drift EDX (100 mm^2^ area each) detectors.

*UV/Vis Spectroscopy:* The absorption of UV/Vis light (200–700 nm) by selected nanoparticle dispersions (CdS/SiO_2_ and CuS/SiO_2_) was measured using a Lambda 18 UV/Vis Spectrometer from Perkin-Elmer after dilution with water to suitable optical densities in cells of 10 mm path length. From the acquired spectra, the wavelength at the onset of significant light absorption was determined and converted into mean radii of the nanoparticle cores via the Brus equation [37,38]. The required values for the band gap energy of the semiconductors as well as the effective masses of their excited electrons and holes were taken from the literature [39,40].

## 3. Results

**Figure 1:**
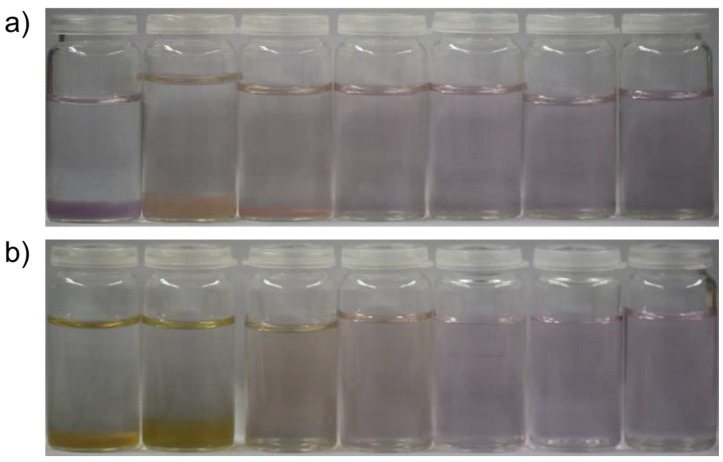
Photographs of samples obtained by mixing equal volumes of 10 mM solutions of CoCl_2_ and (NH_4_)_2_CO_3_, with the latter containing different amounts of dissolved sodium silicate (from left to right): 0, 100, 300, 700, 1000, 1500, and 2000 ppm SiO_2_. The pH was adjusted to (**a**) 9.0 and (**b**) 11.0 after mixing with the addition of HCl and NaOH.

The experiments performed in this work were utterly simple: typically, 10 mM aqueous solutions of chloride salts of the chosen metal cations were mixed in equal volumes with a solution containing the same molar amount of the anion in question, i.e., carbonate or sulfide as their sodium or ammonium salts. To the latter solution, varying concentrations of sodium silicate were added, as obtained by (i) dilution of conventional sodium silicate solution (“water glass”), (ii) dissolution of sodium metasilicate, or (iii) prior hydrolysis of tetraethyl orthosilicate (TEOS) with sodium hydroxide. Whereas solutions containing sodium meta silicate were the easiest to prepare, the hydrolysis of TEOS with constant hydrolysis times led to the best reproducibility. The first experiments were done using sodium metasilicate due to the simplicity of the synthesis process. All the structures shown below can be obtained regardless of which silica source is used. The pH established after mixing the reagents was either left unadjusted or set to 9.0 or 11.0 by dosing aliquots of HCl or NaOH (in order to test the influence of pH on colloidal stability). The effect of added silica on the precipitation of metal carbonates and sulfides from water at ambient conditions becomes evident already from the visual appearance of samples, as illustrated in Figure 1 for the case of cobalt carbonate obtained from CoCl_2_ and NH_4_CO_3_. In the absence of silica and at low concentrations (e.g., 50 ppm SiO_2_ after mixing), relatively large solid particles are nucleated and aggregated to form a flocculated material that sediments to the bottom of the sample container. The color of this material varies as a function of silica concentration and pH, arguably due to a change in primary particle size. In contrast, at final SiO_2_ contents of ≥350 ppm (pH 9.0) or ≥150 ppm (pH 11.0), respectively, no visual precipitation occurs anymore, and homogeneous solutions with a slight violet color are obtained. This suggests that small nanoparticles of CoCO_3_ have formed and remain finely dispersed in their mother medium due to the stabilizing influence of silicate.

Analyses of the size of the species present in the colloidally stable systems by means of dynamic light scattering (DLS) indicate the formation of primary particles with diameters of less than 10 nm, which coexist with a certain fraction of larger entities (typically up to 100 nm; see Table S1 in the Supporting Information (SI)). The measured sizes of the primary particles and their secondary aggregates do not show clear systematic trends with pH and/or silicate concentration, probably as a consequence of different, partly opposing effects: higher levels of pH will increase the supersaturation of the solutions with respect to metal carbonate formation (potentially leading to larger particles), but also enhance the degree of charges at siliceous species [41], which should impede particle aggregation due to electrostatic repulsion. However, the latter effect may also be mitigated or even reversed by association and the bridging of free divalent cations. On the other hand, higher silicate concentrations should be more effective at capturing CoCO_3_ cores at small sizes, but will also favor particle aggregation through polycondensation (which again depends intimately on the pH value).

**Figure 2:**
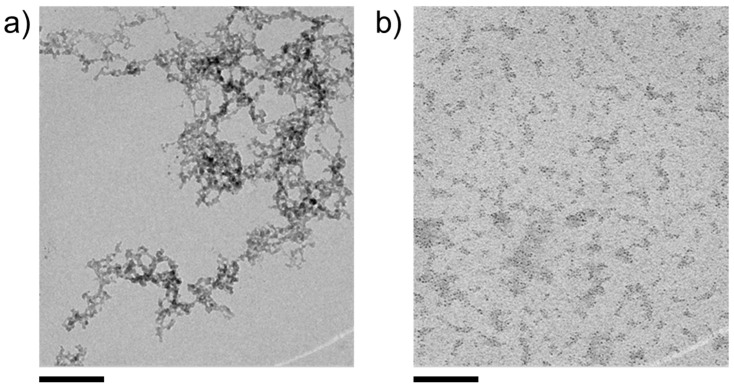
TEM micrographs of typical reaction products obtained from mixtures containing 10 mM each of CoCl_2_, Na_2_CO_3_, and SiO_2_ at native pH. (**a**) Colloidal aggregates formed by cation-induced condensation of silicate species. (**b**) Numerous CoCO_3_ nanodots (dark spots), partially embedded in diffuse silica matrices (areas of lower contrast). Scale bars: 100 nm.

Further insights into the nature of the formed particles can be gained by TEM analyses, as shown in Figure 2 for CoCO_3_, precipitated in the presence of 10 mM SiO_2_ (600 ppm) and isolated from the aqueous mother solution via centrifugation. At this point, it is important to note that the aforementioned ability of divalent metal cations to trigger silica polycondensation by charge screening [41] can lead to the formation of nanosized colloidal species consisting of amorphous silica with some coordinated metal cations but no explicit metal carbonate (or sulfide) components. An example of these colloidal silica structures observed during the precipitation of CdCO_3_ from a silicate-containing solution is shown in Figure 2a. Indeed, such byproducts of the reaction were occasionally found in all the investigated samples, but their amounts relative to metal carbonate (or sulfide) bearing structures were generally low. Even though the dimensions of the latter are similar to the condensed siliceous networks, they can clearly be distinguished based on morphology and texture, as shown in Figure 2b.

**Figure 3:**
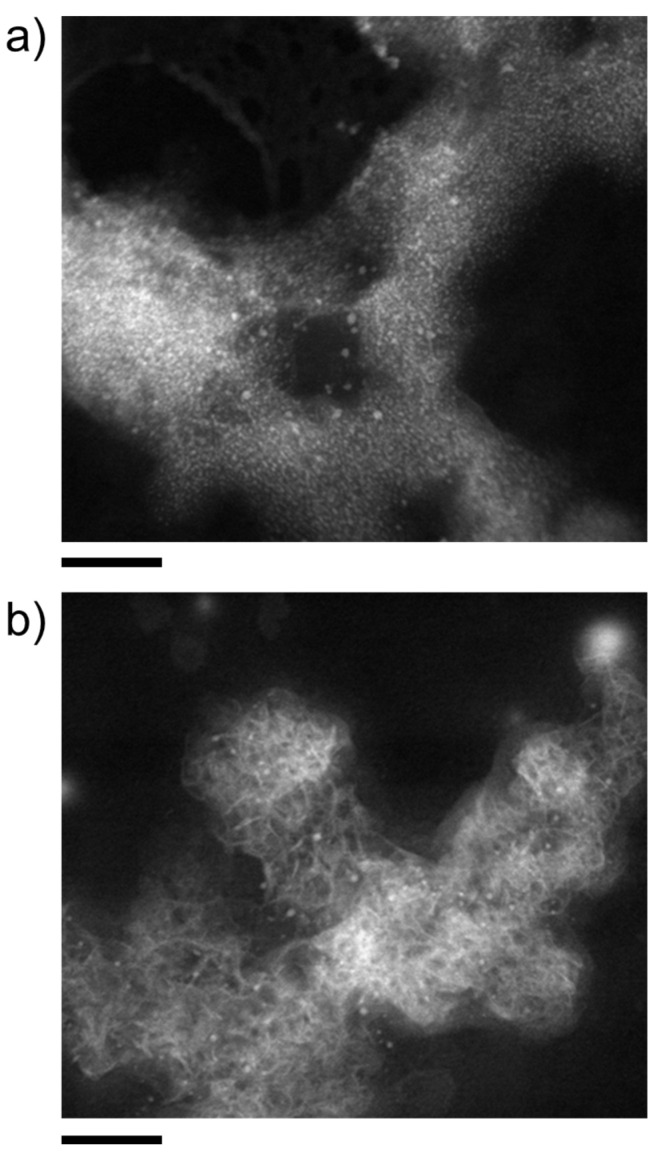
ADF-STEM micrographs of nanostructures obtained by mixing equal volumes of 10 mM solutions of CoCl_2_ and Na_2_CO_3_, with the latter containing 300 ppm (5 mM) SiO_2_ at the native pH of 10.35. Scale bars: 50 nm. (**a**,**b**) show different positions on the grid with differing amount of captured nucleation cores, visible as white dots.

In such images, nanodots of, arguably, CoCO_3_ can clearly be discerned and are found to be embedded in cloud-like domains of lower electron contrast, arguably silica matrices, which interconnect multiple individual cores. In fact, the two types of structure shown in Figure 2 can also exist in mixed forms, for example, colloidal silica aggregates with attached and/or occluded metal carbonate nanodots. Imaging nanodot-rich structures, such as those in Figure 2b, using annular dark-field scanning transmission electron microscopy (ADF-STEM, Figure 3) reveals extended networks (likely formed by forced aggregation upon centrifugation), which appear to consist of a continuous matrix of amorphous silica with multiple embedded CoCO_3_ cores (Figure 3a). This cannot be proven directly as the small particles are destroyed during STEM measurement with high resolution, or are contaminated by carbon impurities during EDX mapping. Individual cores can be discerned at the edges and in the middle of the agglomerates with sizes of a few nanometers, in good agreement with the DLS data (cf. Table S1 in the SI).

Interestingly, the ADF-STEM analyses also reveal the occasional formation of another type of structure, consisting of a network of plate-like particles along with randomly distributed nanodots (Figure 3b). This suggests that in some cases, the stabilization of nanodots by silica is insufficient and allows partial CoCO_3_ crystallization to occur. For both types of structure, the presence of cobalt and carbonate, along with silica, is confirmed by EDX (energy-dispersive X-ray spectroscopy) elemental mapping (see Figure S1 in the SI).

**Figure 4:**
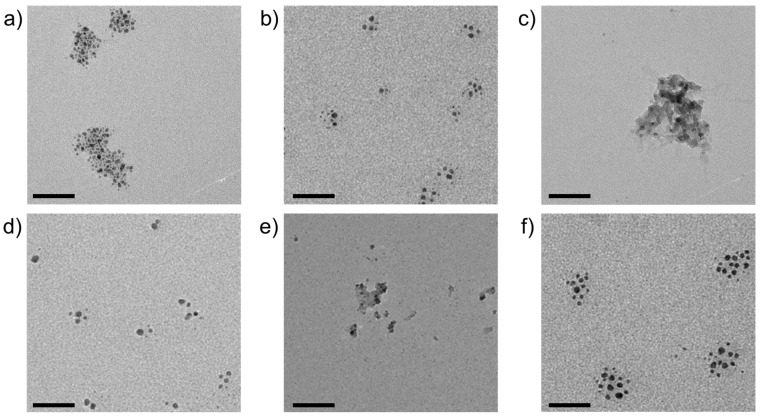
TEM micrographs of silica-coated nanoparticles of (**a**) CdCO_3_, (**b**) CoCO_3_, (**c**) CuCO_3_, (**d**) MnCO_3_, (**e**) NiCO_3,_ and (**f**) ZnCO_3_, obtained by spontaneous co-precipitation from solutions containing 10 mM each of metal chloride, Na_2_CO_3,_ and sodium silicate, at the respective native pH of around 10.5. Scale bars: 50 nm.

Based on the observations made for CoCO_3_ as a function of pH and silicate concentration (cf. Figure 1), a screening of different transition metal cations was performed at fixed conditions of 10 mM metal chloride, 10 mM sodium carbonate, and 10 mM sodium silicate (corresponding to ca. 600 ppm SiO_2_) at the respective native pH. Representative TEM images of samples prepared by direct blotting of diluted aqueous solutions on carbon-filmed copper grids are summarized in Figure 4. It is evident that cores with high electron contrast and diameters below 10 nm were formed with all metal cations investigated. Interestingly, the degree of aggregation can be classified into three groups, where the nanoparticles exist (i) mostly separated from one another with low affinity to aggregate (Co^2+^ (pK_A_ = 9.7) and MnCO_3_ (pK_A_ = 10.6)), (ii) as loose agglomerates in which individual particles retain certain mutual distances (Cd^2+^ (pK_A_ = 10.1)and Zn^2+^ (pK_A_ = 9.0)), or (iii) embedded in a matrix of silica that encompasses multiple cores (Cu^2+^ (pK_A_ = 7.5) and Ni^2+^ (pK_A_ = 9.8)).

The influence of metal cations on the degree of silica polycondensation around nucleated and growing metal carbonate nanoparticles should depend on (i) the concentration of cations remaining in solution after initial nanoparticle formation and (ii) their affinity to bind to silicate species and catalyze condensation via charge screening and bridging. The latter effect may be expected to scale with cation acidity; however, there is no clear relationship between the reported pK_A_ values (as given above [42]) and the observed aggregation behavior, likely due to a convolution of cation properties and concentration. The amount of residual dissolved cations after nucleation is, in turn, difficult to estimate. One possible approach would be to use the solubility products; however, reported solubilities for the respective bulk mineral likely do not apply to nanometric particles [43] and per se, the nature of phases formed in the present experiments is not unambiguous. For example, precipitation of Cu^2+^ should result in basic copper carbonate (i.e., Cu_2_CO_3_(OH)_2_ instead of CuCO_3_) under the given conditions [44]. Based on the available data, we can, thus, only speculate about the actual composition of the cores and the effects underlying the observed differences in nanoparticle aggregation and silica polycondensation. Nonetheless, it can safely be concluded that the presence of silica during precipitation leads to drastic miniaturization of the formed solid phase, yielding nanodots with typical diameters in the range of 3–20 nm, as measured from TEM images and summarized in Table 1.

**Figure 5:**
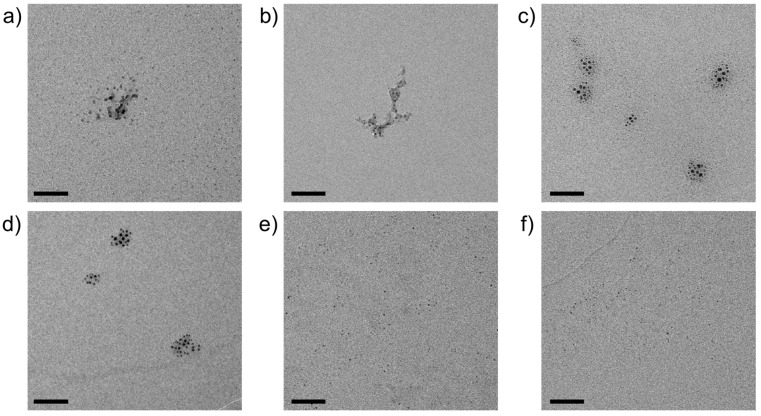
TEM micrographs of oxide nanoparticles obtained by calcination of silica-coated precursors of (**a**) CdCO_3_, (**b**) CoCO_3_, (**c**) CuCO_3_, (**d**) MnCO_3_, (**e**) NiCO_3_, and (**f**) ZnCO_3_, as shown in Figure 4. Note that numerous individual nanodots can be observed across the entire fields of view in (**e**,**f**). Scale bars: 50 nm.

In a subsequent step, the carbonate (and/or hydroxocarbonate) particles obtained via the mixing of metal chloride and sodium carbonate/silicate solutions were calcined according to procedures described in the literature for the respective (hydroxo)carbonate phases [30,31,32,33,34], in order to convert the cores of the nanodots into (catalysis-relevant) oxides. Figure 5 provides an overview of representative TEM micrographs showing typical nanostructures resulting from thermal treatment. Nanodots were observed after calcination for all metal cations investigated, yet again with different degrees of aggregation and silica-mediated crosslinking. Here, well-dispersed individual nanoparticles occur for oxides derived from NiCO_3_, ZnCO_3_, and (to a lesser extent) CdCO_3_. At the same time, loose agglomerates of multiple cores characterize samples obtained via calcination of CuCO_3_ and MnCO_3_, and the most pronounced embedding in remaining silica matrices is observed for CoCO_3_. As expected, the average core size decreases upon calcination, with relative degrees of shrinkage (due to decarboxylation and/or dehydration) ranging from 12 to 47% (cf. Table 1).

As in the case of the carbonate precursor systems (cf. Figure 3), stabilization by silica proved to be sufficient to protect most of the nanodots against strong agglomeration and/or crystallization, but failed in singular instances, as shown for the product of CoCO_3_ calcination by Figure S2 in the SI. Indeed, TEM and ADF-STEM analyses reveal the local formation of cobalt oxide crystals with sizes in the range of 100–200 nm. Their crystallinity could be confirmed by electron diffraction (Figure S3 in the SI). Although such recrystallization events were generally scarce and only occurred upon aging in air, further studies will have to be performed to improve the robustness of the process.

**Figure 6:**
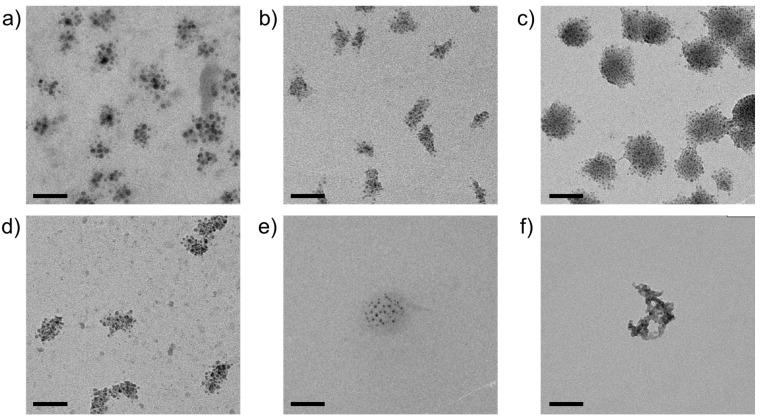
TEM micrographs of silica-coated nanoparticles of (**a**) CdS, (**b**) CoS, (**c**) CuS, (**d**) MnS, (**e**) NiS, and (**f**) ZnS, obtained by spontaneous co-precipitation from solutions containing 10 mM each of metal chloride, Na_2_S, and sodium silicate, at the respective native pH. Scale bars: 50 nm.

The approach described above for metal carbonates can be used in complete analogy to prepare fairly well-defined nanostructures of metal sulfides, as illustrated in Figure 6. While the size of the formed cores is comparable to the corresponding (hydroxo)carbonates (as determined from TEM images, cf. Table 1, and confirmed by UV-Vis measurements, see Figure S4 in the SI), the aggregation behavior again shows interesting differences. For CdS, CoS, CuS, and MnS, multiple primary nanodots assembled into larger secondary structures (typically 20–50 nm in diameter), with morphologies and aggregation numbers that are quite uniform for a given metal cation but differ significantly from cation to cation. In all of these cases, individual cores maintain certain distances from one another within the aggregates and seem to be interspersed by a matrix of lower electron density, most likely consisting of amorphous silica. The “cloud-like” appearance of these secondary structures suggests a self-limiting aggregation process, driven and arrested by the amount of residual free metal cations and their influence on local silica polycondensation.

## 4. Discussion

The data collected in this work provides compelling evidence that direct spontaneous co-precipitation of various transition metal cations with carbonate and sulfide ions from silicate-rich solutions can lead to the spontaneous formation of primary nanoparticles with typical sizes below 5 nm. These self-assembled nanodots are obtained as a consequence of the miniaturizing influence of silica, likely facilitated by local co-mineralization, as illustrated in Scheme 1. The fundamental feasibility of reducing the dimensions of nanoparticles to such small sizes by chemically coupled processes has previously been reported for clusters of calcium carbonate [28] and was proposed to occur intrinsically during the growth of self-assembled silica/barium carbonate structures [45]. Very recently, we could show that the same principle may be used to prepare silica-coated quantum dots of cadmium as well as zinc and mixed cadmium/zinc sulfides and selenides in simple one–pot procedures [29]. In this regard, the experimental results of the present study can be considered an extension of the approach toward a broader range of functional materials, thus, confirming the initial hypothesis that pH-based chemical coupling may serve as a general strategy for synthesizing (silica-coated) nanoparticles. While (hydroxo)carbonate (cf. Figure 4) and sulfide (cf. Figure 6) phases of different (transition) metals are directly accessible through spontaneous co-precipitation at ambient conditions, corresponding oxides can be obtained via thermal post-treatment of the carbonate nanoparticles (cf. Figure 5). In principle, both oxides and hydroxides should also be suitable anions to incur pH-based chemical coupling due to the release of protons upon mineralization, as exemplified below for the cases of zinc and manganese:

(4) $${\mathrm{Zn}}^{2+}{+2\;H}_{2}O\;\to \;\mathrm{Zn}{\left(\mathrm{OH}\right)}_{2}\;\;+2\;H^{+}\to \;\mathrm{ZnO}+H_{2}O+2\;H^{+}$$

(5) $$2{\;\mathrm{MnO}}_{4}^{-}{+3\;\mathrm{Mn}}^{2+}{+2\;H}_{2}O\;\to \;5{\;\mathrm{MnO}}_{2}+4\;H^{+}$$

However, tests performed so far have shown that the absence of a buffer equilibrium (as provided by both carbonates and sulfides) results in uncontrolled conditions, which do not allow uniform nanoparticles to be formed by direct co-precipitation. Additionally, this direct reaction path is not suitable for all transition metals investigated. Therefore, we chose to prepare the oxides by calcinating the corresponding carbonate salts for the present work.

Although further characterization of the obtained nanodots is certainly required (especially concerning the structure of the cores and the presence of a silica shell around them), the observed miniaturization and presumed coating of the resulting particles by silicate species should have the following beneficial effects on the properties of the nanomaterials:

Enlargement of their specific surface area.

Protection of the surface of individual nanodots through thin layers of silica (e.g., to prevent oxidation in the air or to shield dipole–dipole interactions in case of magnetic particles) while at the same time, the silica layer can still be permeable for solutes (e.g., in catalysis) or gradually release the encapsulated material (e.g., for delivery applications); both are essentially controlled by the thickness of the shell, which can easily be tuned by adjusting the concentration of silica in solution [26].

Higher colloidal stability: as a consequence of their large surface-to-volume ratios, nanosized particles are inherently unstable with a high tendency to aggregate to minimize surface energy; a negatively charged coating (e.g., silica) could enhance Coulomb as well as steric repulsion and, thus, decrease their tendency to agglomerate (cf. Figure 1) [46].

Surface functionalization: another general advantage of silica coatings is the well-established silane chemistry for surface modification; the free silanol groups at the surface can, thus, be chemically modified to afford different functionalities, such as amines or carboxylates [47].

Control over crystallinity and/or polymorphism: as in the case of the alkaline-earth carbonates [26,27,28], rapid encapsulation of early (hydroxo)carbonate/sulfide particles could allow for the isolation and stabilization of otherwise transient precursor phases, for instance, amorphous states.

Particular interest in capturing and preserving transient disordered phases originates from the finding that such materials often show higher catalytic activity than their crystalline counterparts [48,49]. The procedure described herein could, thus, provide easy access to these promising phases and stabilize them against transformation, as shown for, e.g., amorphous calcium and barium carbonate [26,27]. On a more fundamental level, the approach might also be utilized to study crystallization mechanisms in detail with respect to short-lived intermediates [28]. Current work is, therefore, focused on a profound characterization of the obtained nanodots at high resolution as well as testing their potential in selected catalytic applications.

Indeed, there is a vast body of literature available on the synthesis of nanodots, most notably those based on carbon [50], but also different types of transition metal oxides [51,52,53] and their use for catalysis [54,55] and other areas [56,57]. Likewise, previous studies have reported various ways to obtain metal sulfide quantum dots [22] or transition metal oxide nanoparticles [58,59,60,61,62] with a coating of amorphous silica. Compared to all these existing concepts for the preparation of functional core–shell nanoparticles, the present approach is clearly distinguished by its simplicity, along with the versatility in terms of core composition (as highlighted by the results of this study) and shell properties (e.g., thickness, porosity, and condensation of silica layers, still to be demonstrated). Altogether, the described method for nanodot preparation appears attractive as it (i) relies on plain mixing of two solutions (with no need for the use of complex templates or exfoliation procedures); (ii) takes place in a one–pot reaction utilizing self-assembly (in contrast to many other reported protocols for core–shell structures, where nanoparticles are typically prepared first and subsequently coated with silica in a second step, e.g., via the Stöber process); (iii) does not require any specific organic additives such as capping or exfoliation agents; (iv) can be carried out in water (i.e., no organic solvents needed) at standard pressure and room temperature: and (v) gives straightforward access to a variety of nanodots through the reaction of (transition) metal cations with either sulfides or carbonates, whereby secondary calcination of the latter can be used to transform the initially obtained compositions into their corresponding oxides. Given these advantages, simple co-precipitation from silicate-containing solutions seems to be a promising strategy for the sustainable synthesis of functional nanostructures, warranting further detailed studies of the obtained products (e.g., by high-resolution TEM) and their potential in catalytic processes and other applications.

## 5. Conclusions

In the present work, we have performed a series of straightforward precipitation experiments in which selected transition metal chlorides were reacted with sulfides and carbonates under the influence of dissolved silicate at elevated pH in a purely aqueous system at ambient conditions. Phenomenological characterization of the obtained products utilizing conventional transmission electron microscopy showed that this simple procedure resulted in defined nanometric structures containing nanodots of the desired metal carbonate/sulfide coated in a silica matrix and other predominantly silicate-containing structures. Apart from offering a possible alternative to existing experimental routines for synthesizing and coating nanoparticles with silica, our approach has several advantages compared to others. Most importantly, our syntheses are simple and nevertheless offer large degrees of freedom in terms of their output. They only involve one reaction step, and no elaborate precursors [63] or sacrificial templates [64] have to be prepared in advance. Instead, the process solely relies on mixing aqueous solutions of commercially available reactants in a simple one–pot route. Additionally, all experiments can be conducted in ambient conditions, i.e., standard pressure and room temperature. The only two parameters to be modified are pH and the concentration of reactants, both being easily controlled by an automated titration setup. Even further, by adjusting these two handles carefully, the thickness and permeability of the silica shell, as well as the size of the coated nanoparticles, can be tuned. The spontaneous encapsulation of functional (e.g., catalytically active) nanoparticles in layers of silica is expected to improve the performance of corresponding materials by increasing the specific surface area, providing colloidal stability, hindering aggregation, and offering the possibility of straightforward chemical modification. Particles produced in this simple, unelaborate spontaneous coprecipitation can be used in a variety of applications. Particularly in areas such as catalysis or bioimaging, where usually particles produced in elaborate sol–gel processes are used. For such applications, however, individual optimization is still required. Nevertheless, the simplicity of the synthesis combines many individual steps of other syntheses and significantly accelerates the process of nanoparticle production.

## Figures and Tables

**Table 1:** Mean diameters (d) determined from TEM micrographs like those shown in Figure 4, Figure 5 and Figure 6 for silica-stabilized nanoparticles of different metal cations with different anions. Carbonate and sulfide samples were obtained via spontaneous co-precipitation from solutions containing 10 mM each of metal chloride, sodium silicate, and sodium carbonate or sodium sulfide. Oxide particles were formed upon calcination of their carbonate counterparts. For each system, a statistically relevant number (n) of nanoparticles was analyzed, giving reliable mean diameters with corresponding standard deviations. Note that calcination leads to a decrease in the average size, with relative changes ranging from 12 (Cu^2+^) to 47% (Zn^2+^).

Metal Cation	dCarbonate/nm	dOxide/nm	dSulfide/nm
Cd^2+^	4.8 ± 1.1 (n = 90)	3.5 ± 0.9 (n = 55)	5.3 ± 2.2 (n = 68)
Co^2+^	4.1 ± 1.2 (n = 140)	2.8 ± 0.7 (n = 70)	4.4 ± 1.5 (n = 47)
Cu^2+^	4.8 ± 1.1 (n = 10)	4.3 ± 1.4 (n = 86)	5.5 ± 1.3 (n = 29)
Mn^2+^	5.7 ± 1.7 (n = 70)	3.7 ± 1.0 (n = 230)	4.5 ± 1.7 (n = 40)
Ni^2+^	4.4 ± 1.1 (n = 21)	2.9 ± 0.7 (n = 107)	4.7 ± 1.0 (n = 45)
Zn^2+^	4.7 ± 1.4 (n = 90)	2.5 ± 0.5 (n = 133)	3.3 ± 0.7 (n = 38)

## Data Availability

Data is contained within the article or Supplementary Materials.

## Supplementary Materials

The following supporting information can be downloaded at: https://www.mdpi.com/article/10.3390/nano14242054/s1.
