# Supplementary material for: Nanodots of Transition Metal Sulfides, Carbonates, and Oxides Obtained Through Spontaneous Co-Precipitation with Silica
Source: Nanomaterials (Basel). 2024 Dec 23;14(24):2054. doi: 10.3390/nano14242054 (PMC11678075; doi:10.3390/nano14242054)
Supplement: Supplementary file 1 [file nanomaterials-14-02054-s001.zip › nanomaterials-3293343-supplementary.pdf]

# Nanodots of Transition Metal Sulfides, Carbonates, and Oxides Obtained Through Spontaneous Co-Precipitation with Silica

*Bastian Rödig *, Diana Funkner, Thomas Frank, Ulrich Schürmann, Julian Rieder, Lorenz Kienle, Werner Kunz * and Matthias Kellermeier **

During growth of transition metal sulfides and carbonates (red) in aqueous silicate solutions, the pH at active fronts (green) is lowered and triggers local polycondensation of silica (blue). This prevents the particles from growing larger than few nanometers and stabilizes them against agglomeration. The described method thus gives simple access to nanodots of various functional materials, which may be converted into the corresponding oxides by secondary thermal treatment.

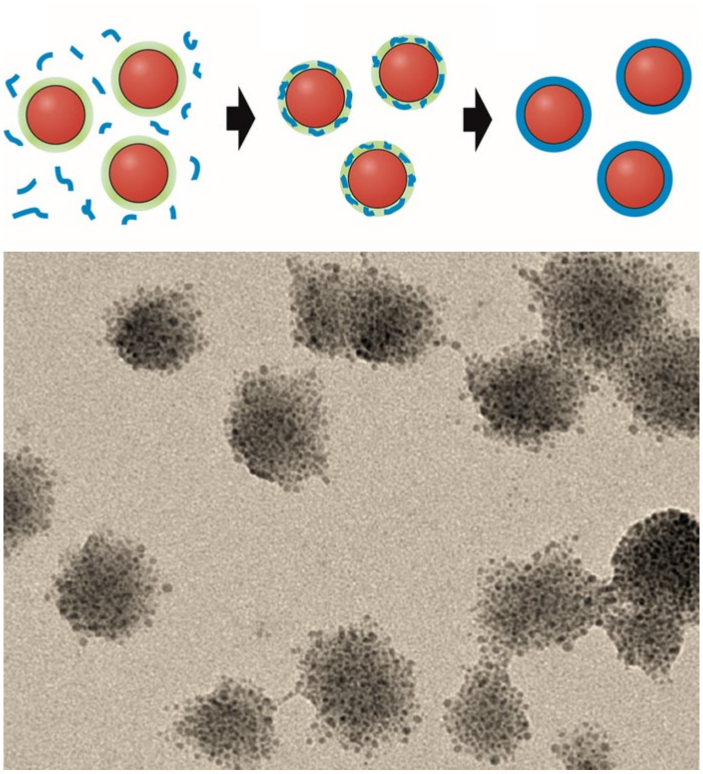

# Supporting Information

Nanodots of Transition Metal Sulfides, Carbonates and Oxides Obtained by Spontaneous Co-Precipitation with Silica

Bastian Rödig, Diana Funkner, Thomas Frank, Ulrich Schürmann, Dominik Zahnweh, Julian Rieder, Lorenz Kienle, Werner Kunz* and Matthias Kellermeier*

**Table S1.** Apparent particle and aggregate sizes determined by dynamic light scattering for $CoCO_3$ dispersions obtained by mixing equal volumes of 10 mM $CoCl_2$ and $(NH_4)_2CO_3$ solutions in the presence of different amounts of silicate at varying final pH. Average diameters were derived from the DLS data using the cumulants method[S1] and the CONTIN algorithm,[S2] which

gives apparent sizes for both the primary particles and their aggregates. Note that corresponding analyses of samples containing 0 or 50 ppm $SiO_2$ did not yield meaningful results due to the formation of large particles, which subsequently aggregated and sedimented (as shown in Figure 1 of the main text).

| $[SiO_2]$ / ppm | pH | DLS (Cumulants) d / nm | DLS (CONTIN) d / nm |
|---|---|---|---|
| 150 | 8.4 (native) | n.d. | 29.4 (100 %) |
| | 9.0 | 65.7 | 1.6 (97.2 %)<br>8.2 (2.8 %) |
| | 11.0 | 52.7 | 1.2 (98.4 %)<br>15.6 (1.6 %) |
| 350 | 9.0 | 43.9 | 8.1 (100%) |
| | 9.2 (native) | 48.6 | 5.6 (96.8 %)<br>19.2 (3.2 %) |
| | 11.0 | 36.0 | 6.8 (100 %) |
| 500 | 9.0 | 41.4 | 6.5 (99.6 %)<br>18.4 (0.4 %) |
| | 9.6 (native) | 51.9 | 12.3 (99.7 %)<br>44.3 (0.3 %) |
| | 11.0 | 19.4 | 2.4 (97.3 %)<br>8.6 (2.7 %) |
| 750 | 9.0 | 12.4 | 3.0 (100 %) |
| | 10.0 (native) | 10.8 | 4.2 (98.3 %)<br>16.3 (2.7 %) |

| | | | |
|---|---|---|---|
| | 11.0 | 40.2 | 1.2 (99.3 %)<br>13.6 (0.7 %) |
| | 9.0 | 7.6 | 3.0 (100 %) |
| 1000 | 10.3 (native) | 36.1 | 3.0 (98.8 %)<br>12.1 (1.2 %) |
| | 11.0 | 26.9 | 3.5 (99.2 %)<br>14.2 (0.8 %) |

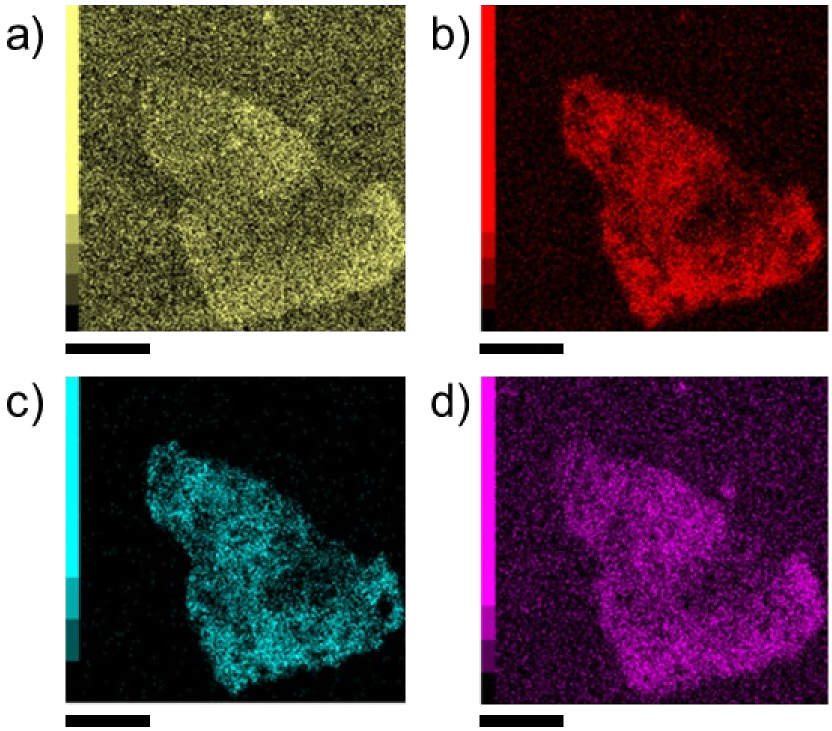


**Figure S1.** Elemental mapping of nanostructures as shown by Figure 3 in the main text: a) carbon, b) oxygen, c) cobalt and d) silicon. Scale bars: 250 nm.

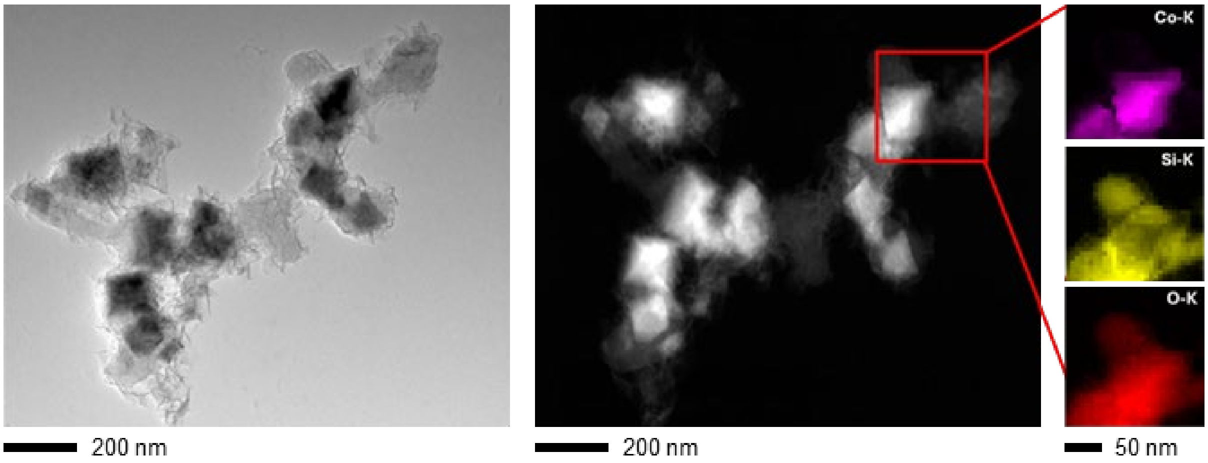


**Figure S2.** TEM (left) and HAADF-STEM (middle) micrographs of nanostructures formed upon calcination of silica-stabilized cobalt carbonate precursors as shown in Figure 4b of the main text and obtained by mixing equal volumes of 10 mM solutions of $CoCl_2$ and $Na_2CO_3$, with the latter containing 600 ppm (10 mM) $SiO_2$. Elemental maps (right) confirm the presence of Co, Si and O, consistent with silica-coated cobalt oxide nanocrystals.

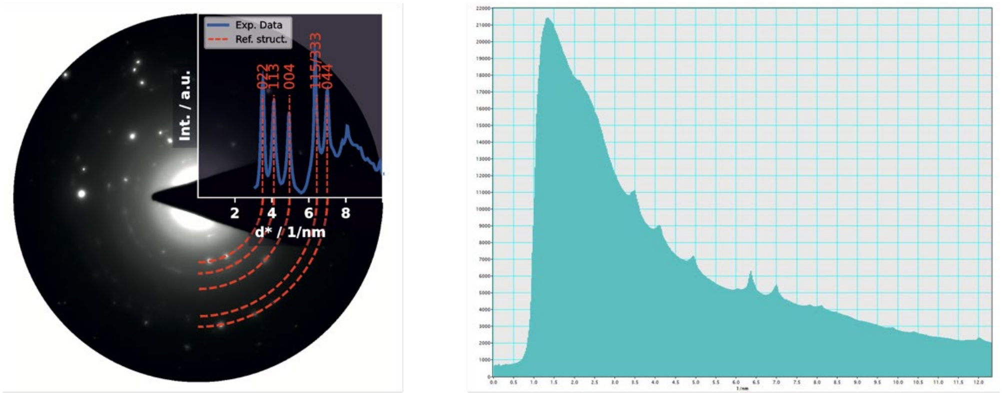


**Figure S3.** Electron diffraction (ED) pattern (left) and the corresponding diffractogram obtained by radial integration (right) from the nanostructures shown in Figure S2. The observed reflections suggest a spinel structure, probably $Co_3O_4$.

.

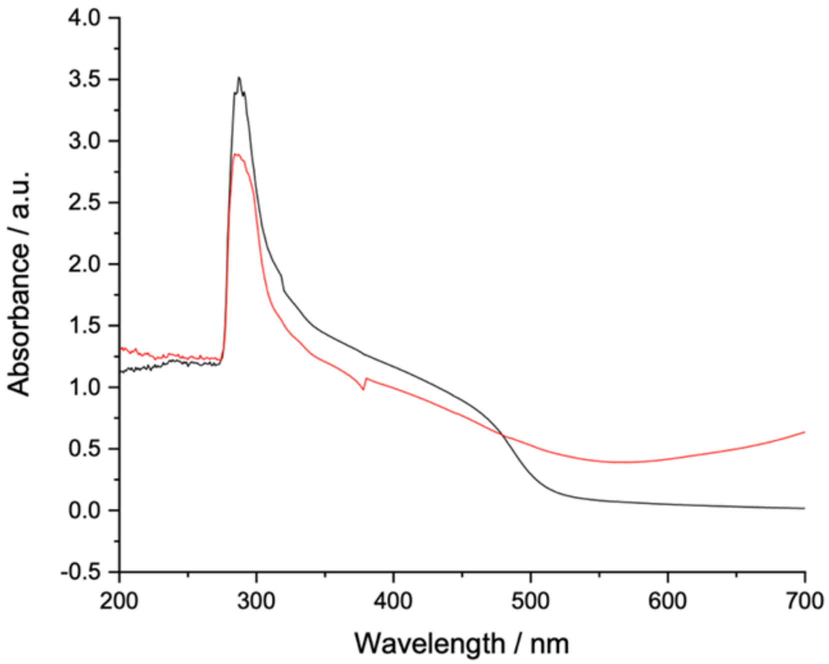


**Figure S2.** UV/Vis spectra of CdS (black) and CuS (red) particles formed from aqueous solutions containing 10 mM each of transition metal chloride, sodium sulfide, and sodium silicate. Using the Brus equation with an onset of absorption of 312 nm for CdS and 304 nm for CuS, diameters of 2.51 nm and 2.56 nm can be calculated, respectively.
